# Supplementary material for: Unveiling the Bioactive Potential of the Invasive Jellyfish Phyllorhiza punctata Through Integrative Transcriptomic and Proteomic Analyses
Source: Biomolecules. 2025 Aug 4;15(8):1121. doi: 10.3390/biom15081121 (PMC12383608; doi:10.3390/biom15081121)
Supplement: Supplementary file 1 [file biomolecules-15-01121-s001.zip › Suplementary Material/FileS3/PEPFOLD4/XP_065059971_5522/clusters.html]

 PEP-FOLD clusters


Download archive of all models  here   

|  |  |  |
| --- | --- | --- |
| # | file | sOPEP |
| # cluster 1 - Size: 28 |  |  |
|  | XP\_065059971\_5522-model1 | -4.64891 |
|  | XP\_065059971\_5522-model1.1 | -4.64891 |
|  | XP\_065059971\_5522-model1.2 | -4.64891 |
|  | XP\_065059971\_5522-model1.3 | -4.17045 |
|  | XP\_065059971\_5522-model1.4 | -3.96287 |
|  | XP\_065059971\_5522-model1.5 | -3.8958 |
|  | XP\_065059971\_5522-model1.6 | -3.83212 |
|  | XP\_065059971\_5522-model1.7 | -3.82804 |
|  | XP\_065059971\_5522-model1.8 | -3.73135 |
|  | XP\_065059971\_5522-model1.9 | -3.66222 |
|  | XP\_065059971\_5522-model1.10 | -3.61044 |
|  | XP\_065059971\_5522-model1.11 | -3.6011 |
|  | XP\_065059971\_5522-model1.12 | -3.56413 |
|  | XP\_065059971\_5522-model1.13 | -3.50747 |
|  | XP\_065059971\_5522-model1.14 | -3.50747 |
|  | XP\_065059971\_5522-model1.15 | -3.47572 |
|  | XP\_065059971\_5522-model1.16 | -3.38885 |
|  | XP\_065059971\_5522-model1.17 | -3.38241 |
|  | XP\_065059971\_5522-model1.18 | -3.38241 |
|  | XP\_065059971\_5522-model1.19 | -3.37341 |
|  | XP\_065059971\_5522-model1.20 | -3.36792 |
|  | XP\_065059971\_5522-model1.21 | -3.34284 |
|  | XP\_065059971\_5522-model1.22 | -3.28551 |
|  | XP\_065059971\_5522-model1.23 | -3.26429 |
|  | XP\_065059971\_5522-model1.24 | -3.17433 |
|  | XP\_065059971\_5522-model1.25 | -3.05411 |
|  | XP\_065059971\_5522-model1.26 | -2.88323 |
|  | XP\_065059971\_5522-model1.27 | -2.4424 |
| # cluster 2 - Size: 11 |  |  |
|  | XP\_065059971\_5522-model2 | -4.18021 |
|  | XP\_065059971\_5522-model2.1 | -4.18021 |
|  | XP\_065059971\_5522-model2.2 | -3.95654 |
|  | XP\_065059971\_5522-model2.3 | -3.72436 |
|  | XP\_065059971\_5522-model2.4 | -3.64232 |
|  | XP\_065059971\_5522-model2.5 | -3.40912 |
|  | XP\_065059971\_5522-model2.6 | -3.2413 |
|  | XP\_065059971\_5522-model2.7 | -3.08047 |
|  | XP\_065059971\_5522-model2.8 | -3.05706 |
|  | XP\_065059971\_5522-model2.9 | -2.59529 |
|  | XP\_065059971\_5522-model2.10 | -2.56741 |
| # cluster 3 - Size: 16 |  |  |
|  | XP\_065059971\_5522-model3 | -4.05553 |
|  | XP\_065059971\_5522-model3.1 | -4.00899 |
|  | XP\_065059971\_5522-model3.2 | -3.79221 |
|  | XP\_065059971\_5522-model3.3 | -3.67895 |
|  | XP\_065059971\_5522-model3.4 | -3.42957 |
|  | XP\_065059971\_5522-model3.5 | -3.40533 |
|  | XP\_065059971\_5522-model3.6 | -3.38219 |
|  | XP\_065059971\_5522-model3.7 | -3.34877 |
|  | XP\_065059971\_5522-model3.8 | -3.32997 |
|  | XP\_065059971\_5522-model3.9 | -3.31893 |
|  | XP\_065059971\_5522-model3.10 | -3.27421 |
|  | XP\_065059971\_5522-model3.11 | -3.21988 |
|  | XP\_065059971\_5522-model3.12 | -3.11961 |
|  | XP\_065059971\_5522-model3.13 | -3.03838 |
|  | XP\_065059971\_5522-model3.14 | -2.9925 |
|  | XP\_065059971\_5522-model3.15 | -2.84876 |
| # cluster 4 - Size: 3 |  |  |
|  | XP\_065059971\_5522-model4 | -3.36283 |
|  | XP\_065059971\_5522-model4.1 | -3.25088 |
|  | XP\_065059971\_5522-model4.2 | -3.09165 |
| # cluster 5 - Size: 2 |  |  |
|  | XP\_065059971\_5522-model5 | -3.31026 |
|  | XP\_065059971\_5522-model5.1 | -3.07689 |
| # cluster 6 - Size: 2 |  |  |
|  | XP\_065059971\_5522-model6 | -2.90078 |
|  | XP\_065059971\_5522-model6.1 | -2.58722 |
| # cluster 7 - Size: 2 |  |  |
|  | XP\_065059971\_5522-model7 | -2.8996 |
|  | XP\_065059971\_5522-model7.1 | -2.52734 |
| # cluster 8 - Size: 3 |  |  |
|  | XP\_065059971\_5522-model8 | -2.86585 |
|  | XP\_065059971\_5522-model8.1 | -2.85841 |
|  | XP\_065059971\_5522-model8.2 | -2.58246 |
| # cluster 9 - Size: 1 |  |  |
|  | XP\_065059971\_5522-model9 | -2.53789 |
| # cluster 10 - Size: 1 |  |  |
|  | XP\_065059971\_5522-model10 | -2.53237 |
| # cluster 11 - Size: 7 |  |  |
|  | XP\_065059971\_5522-model11 | -2.51755 |
|  | XP\_065059971\_5522-model11.1 | -2.47432 |
|  | XP\_065059971\_5522-model11.2 | -2.4725 |
|  | XP\_065059971\_5522-model11.3 | -2.44471 |
|  | XP\_065059971\_5522-model11.4 | -2.43877 |
|  | XP\_065059971\_5522-model11.5 | -2.43306 |
|  | XP\_065059971\_5522-model11.6 | -2.40292 |
| # cluster 12 - Size: 4 |  |  |
|  | XP\_065059971\_5522-model12 | -2.45739 |
|  | XP\_065059971\_5522-model12.1 | -2.45536 |
|  | XP\_065059971\_5522-model12.2 | -2.41881 |
|  | XP\_065059971\_5522-model12.3 | -2.39907 |
| # cluster 13 - Size: 2 |  |  |
|  | XP\_065059971\_5522-model13 | -2.44906 |
|  | XP\_065059971\_5522-model13.1 | -2.42269 |
| # cluster 14 - Size: 3 |  |  |
|  | XP\_065059971\_5522-model14 | -2.44516 |
|  | XP\_065059971\_5522-model14.1 | -2.43921 |
|  | XP\_065059971\_5522-model14.2 | -2.43787 |
| # cluster 15 - Size: 1 |  |  |
|  | XP\_065059971\_5522-model15 | -2.43982 |
| # cluster 16 - Size: 4 |  |  |
|  | XP\_065059971\_5522-model16 | -2.43786 |
|  | XP\_065059971\_5522-model16.1 | -2.43476 |
|  | XP\_065059971\_5522-model16.2 | -2.43476 |
|  | XP\_065059971\_5522-model16.3 | -2.39767 |
| # cluster 17 - Size: 4 |  |  |
|  | XP\_065059971\_5522-model17 | -2.41482 |
|  | XP\_065059971\_5522-model17.1 | -2.4059 |
|  | XP\_065059971\_5522-model17.2 | -2.38853 |
|  | XP\_065059971\_5522-model17.3 | -2.37985 |
| # cluster 18 - Size: 1 |  |  |
|  | XP\_065059971\_5522-model18 | -2.40935 |
| # cluster 19 - Size: 1 |  |  |
|  | XP\_065059971\_5522-model19 | -2.40409 |
| # cluster 20 - Size: 2 |  |  |
|  | XP\_065059971\_5522-model20 | -2.39772 |
|  | XP\_065059971\_5522-model20.1 | -2.38133 |
| # cluster 21 - Size: 1 |  |  |
|  | XP\_065059971\_5522-model21 | -2.38887 |
| # cluster 22 - Size: 1 |  |  |
|  | XP\_065059971\_5522-model22 | -2.38761 |
|  |  |  |
